# Assessment of ventriculo-peritoneal (VP) shunt malfunction in an in vitro model of artificial CSF flow: influence of CSF protein concentration, CSF contamination and time of shunt incubation

**DOI:** 10.1186/2045-8118-12-S1-P60

**Published:** 2015-09-18

**Authors:** Thomas Joseph Zwimpfer, Matthew Joseph Zwimpfer

**Affiliations:** 1 Vancouver Gen. Hosp., U. of British Columbia, Canada; 2 School of Engineering & Applied Science, U. of Pennsylvania, USA

## Introduction

Experiments were carried out in an In Vitro model of CSF flow through VP shunts (Medtronic Inc) to investigate the influence of three factors on shunt function: 1) CSF protein concentration; 2) CSF contamination and; 3) Time of shunt incubation in the artificial CSF.

## Methods

Two components of a shunt were tested separately: 1) Ventricular catheter and; 2) Medium pressure, non-programmable valve connected to a peritoneal catheter. In the “sterile” artificial CSF groups, each shunt was incubated in one of six different sterile solutions of varying protein conc. (0.5 g/l - normal and 1.0, 2.0, 5.0, 10.0 and 20 g/L), prepared by adding egg protein (“Naturegg” Egg Whites; 1g/9ml) to normal saline, and incubated in sterile artificial CSF for periods of 0, 7, 14 and 46 days.

A “Contaminated” group of shunts were similarly prepared but exposed to skin microbes by handling them with ungloved hands and incubating them in contaminated artificial CSF. The same contaminated CSF solutions were used over the total 46 days of incubation but only 3 different protein conc. (0.5, 5.0 and 20.0 g/L) were prepared.

Both the sterile and contaminated shunts were tested after four different incubation periods (0, 7, 14, 46 days). Each of the two shunt components (1. Ventricular and; 2. Valve/peritoneal catheter) were tested separately by connecting them with I.V. tubing to a manometer, all filled with the test CSF. The manometer was prefilled to a height of 30 cm H20 and CSF was allowed to flow through each shunt component. The elapsed time (s) was recorded when the CSF passed each 5cm increment from 30 to 0 cm H20 or until flow stopped above 0 cm. On a few occasions, CSF did not drop from the 30 cm level. This “shunt blockage” occurred only when testing the valve / peritoneal catheter complex. Height of CSF column (cm) was plotted against time and the best linear fit determined for each data set. The slope of each curve approximated the average rate of CSF flow (cm/s).

## Results

In this In Vitro model, CSF protein of 5g/L or higher was the threshold level to adversely affect CSF flow through the valve/peritoneal catheter component: Incubation for 46 days in a protein conc. of 10 g/L or higher, resulted in a 75% decrease in CSF flow rate (.07 cm/s) and an elevated closing pressure of 11 cm H20, compared to testing at day 0 in 0.5 g/L (rate = .30 cm/s; CP = 6 cm H20). Microbial contamination did further slow flow but only after 46 days incubation in CSF with a protein conc. of greater then 5 g/L. Absence of cellular and humoral mediators of inflammation in this artificial CSF could be addressed in future studies by using CSF from patients with proven shunt infection. Flow through the ventricular catheter was not affected in any situation in this model.

